# Bare-Bones Teaching-Learning-Based Optimization

**DOI:** 10.1155/2014/136920

**Published:** 2014-06-10

**Authors:** Feng Zou, Lei Wang, Xinhong Hei, Debao Chen, Qiaoyong Jiang, Hongye Li

**Affiliations:** ^1^School of Computer Science and Engineering, Xi'an University of Technology, Xi'an 710048, China; ^2^School of Physics and Electronic Information, Huaibei Normal University, Huaibei 235000, China

## Abstract

Teaching-learning-based optimization (TLBO) algorithm which simulates the teaching-learning process of the class room is one of the recently proposed swarm intelligent (SI) algorithms. In this paper, a new TLBO variant called bare-bones teaching-learning-based optimization (BBTLBO) is presented to solve the global optimization problems. In this method, each learner of teacher phase employs an interactive learning strategy, which is the hybridization of the learning strategy of teacher phase in the standard TLBO and Gaussian sampling learning based on neighborhood search, and each learner of learner phase employs the learning strategy of learner phase in the standard TLBO or the new neighborhood search strategy. To verify the performance of our approaches, 20 benchmark functions and two real-world problems are utilized. Conducted experiments can been observed that the BBTLBO performs significantly better than, or at least comparable to, TLBO and some existing bare-bones algorithms. The results indicate that the proposed algorithm is competitive to some other optimization algorithms.

## 1. Introduction

Many real-life optimization problems are becoming more and more complex and difficult with the development of scientific technology. So how to resolve these complex problems in an exact manner within a reasonable time cost is very important. The traditional optimization algorithms are difficult to solve these complex nonlinear problems. In recent years, nature-inspired optimization algorithms which simulate natural phenomena and have different design philosophies and characteristics, such as evolutionary algorithms [1–3] and swarm intelligence algorithms [4–7], are a research field which simulates different natural phenomena to solve a wide range of problems. In these algorithms the convergence rate of the algorithm is given prime importance for solving real-world optimization problems. The ability of the algorithms to obtain the global optima value is one aspect and the faster convergence is the other aspect.

As a stochastic search scheme, TLBO [8, 9] is a newly population-based algorithm based on swarm intelligence and has characters of simple computation and rapid convergence; it has been extended to the function optimization, engineering optimization, multiobjective optimization, clustering, and so forth [9–17]. TLBO is a parameter-free evolutionary technique and is also gaining popularity due to its ability to achieve better results in comparatively faster convergence time to genetic algorithms (GA) [1], particle swarm optimizer (PSO) [5], and artificial bee colony algorithm (ABC) [6]. However, in evolutionary computation research there have been always attempts to improve any given findings further and further. This work is an attempt to improve the convergence characteristics of TLBO further without sacrificing the accuracies obtained in TLBO and in some occasions trying to even better the accuracies. The aims of this paper are of threefold. First, authors propose an improved version of TLBO, namely, BBTLBO. Next, the proposed technique is validated on unimodal and multimodal functions based on different performance indicators. The result of BBTLBO is compared with other algorithms. Results of both the algorithms are also compared using statistical paired *t*-test. Thirdly, it is applied to solve the real-world optimization problem.

The remainder of this paper is organized as follows. The TLBO algorithm is introduced in Section 2.  Section 3 presents a brief overview of some recently proposed bare-bones algorithms.  Section 4 describes the improved teaching-learning-based optimization algorithm using neighborhood search (BBTLBO).  Section 5 presents the tests on several benchmark functions and the experiments are conducted along with statistical tests. The applications for training artificial neural network are shown in Section 6. Conclusions are given in Section 7.

## 2. Teaching-Learning-Based Optimization

Rao et al. [8, 9] first proposed a novel teaching-learning-based optimization (TLBO) inspired from the philosophy of teaching and learning. The TLBO algorithm is based on the effect of the influence of a teacher on the output of learners in a class which is considered in terms of results or grades. The process of working of TLBO is divided into two parts. The first part consists of “teacher phase” and the second part consists of “learner phase.” The “teacher phase” means learning from the teacher and the “learner phase” means learning through the interaction between learners.

A good teacher is one who brings his or her learners up to his or her level in terms of knowledge. But in practice this is not possible and a teacher can only move the mean of a class up to some extent depending on the capability of the class. This follows a random process depending on many factors. Let *M* be the mean and let *T* be the teacher at any iteration. *T* will try to move mean *M* toward its own level, so now the new mean will be *T* designated as *M*_new_. The solution is updated according to the difference between the existing and the new mean according to the following expression:

(1) $$\begin{array}{c} newX=X+r\times \left(M_{\text{new}}-\text{TF}\times M\right), \end{array}$$

where TF is a teaching factor that decides the value of mean to be changed and *r* is a random vector in which each element is a random number in the range [0,1]. The value of TF can be either 1 or 2, which is again a heuristic step and decided randomly with equal probability as

(2) $$\begin{array}{c} \text{TF}=\text{round}\left[1+\text{rand}\left(0,1\right)\right]. \end{array}$$

Learners increase their knowledge by two different means: one through input from the teacher and the other through interaction between themselves. A learner interacts randomly with other learners with the help of group discussions, presentations, formal communications, and so forth. A learner learns something new if the other learner has more knowledge than him or her. Learner modification is expressed as

(3) $$\begin{array}{c} newX_{i}=\{\begin{array}{cc} X_{i}+r*\left(X_{i}-X_{j}\right) & \text{if}\;f\left(X_{i}\right)<f\left(X_{j}\right)\\ X_{i}+r*\left(X_{j}-X_{i}\right) & \text{otherwise}. \end{array} \end{array}$$

As explained above, the pseudocode for the implementation of TLBO is summarized in Algorithm 1.

## 3. Bare-Bones Algorithm

In this section, we only presented a brief overview of some recently proposed bare-bones algorithms.

### 3.1. BBPSO and BBExp

PSO is a swarm intelligence-based algorithm, which is inspired by the behavior of birds flocking [5]. In PSO, each particle is attracted by its personal best position (*p*_best_) and the global best position (*g*_best_) found so far. Theoretical studies [18, 19] proved that each particle converges to the weighted average of *p*_best_ and *g*_best_:

(4) $$\begin{array}{c} \underset{t\to \infty }{\lim}X_{i}\left(t\right)=\frac{c_{1}\cdot g_{\text{best}}+c_{2}\cdot p_{\text{best}}}{c_{1}+c_{2}}, \end{array}$$

where *c*_1_ and *c*_2_ are two leaning factors in PSO.

Based on the convergence characteristic of PSO, Kennedy [20] proposed a new PSO variant called bare-bones PSO (BBPSO). Bare-bones PSO retains the standard PSO social communication but replaces dynamical particle update with sampling from a probability distribution based on *g*_best_ and *p*_best_*i*__ as follows:

(5) $$\begin{array}{c} x_{i,j}\left(t+1\right)=N\left(\frac{g_{\text{best}}+p_{\text{bes}{\text{t}}_{i,j}}\left(t\right)}{2},\left|g_{\text{best}}-p_{{\text{best}}_{i,j}}\left(t\right)\right|\right), \end{array}$$

where *x*_*i*,*j*_(*t* + 1) is the *j*th dimension of the *i*th particle in the population and *N* represents a Gaussian distribution with mean (*g*_best_ + *p*_best_*i*,*j*__(*t*))/2 and standard deviation |*g*_best_ − *p*_best_*i*,*j*__(*t*)|.

Kennedy [20] proposed also an alternative version of the BBPSO, denoted by BBExp, where (5) is replaced by

(6) xi,j(t+1) ={N(gbest+pbesti,j(t)2,|gbest−pbesti,j(t)|)rand(0,1)>0.5pbesti,j(t)otherwise,

where rand(0,1) is a random value within [0,1] for the *j*th dimension. For the alternative mechanism, there is a 50% chance that the search process is focusing on the previous best positions.

### 3.2. BBDE, GBDE, and MGBDE

Inspired by the BBPSO and DE, Omran et al. [21] proposed a new and efficient DE variant, called bare-bones differential evolution (BBDE). The BBDE is a new, almost parameter-free optimization algorithm that is a hybrid of the bare-bones particle swarm optimizer and differential evolution. Differential evolution is used to mutate, for each particle, the attractor associated with that particle, defined as a weighted average of its personal and neighborhood best positions. For the BBDE, the individual is updated as follows:

(7) xi,j(t+1) ={pi3,j(t)+r2·(xi1,j(t)−xi2,j(t))rand(0,1)>CRpbesti3,j(t)otherwise,

where *i*_1_, *i*_2_, and *i*_3_ are three indices chosen from the set {1,2,…, NP} with *i*_1_ ≠ *i*_2_ ≠ *i*, rand (0, 1) is a random value within [0, 1] for the *j*th dimension, and *p*_*i*,*j*_(*t*) is defined by

(8) $$\begin{array}{c} p_{i,j}\left(t+1\right)=r_{1,j}\cdot p_{{\text{best}}_{i,j}}\left(t\right)+\left(1-r_{2,j}\right)g_{{\text{best}}_{i}}\left(t\right), \end{array}$$

where *p*_best_ and *g*_best_ are personal best position and the global best position, *r*_1,*j*_, is a random value within [0,1] for the *j*th dimension.

Based on the idea that the Gaussian sampling is a fine tuning procedure which starts during exploration and is continued to exploitation, Wang et al. [22] proposed a new parameter-free DE algorithm, called GBDE. In the GBDE, the mutation strategy uses a Gaussian sampling method which is defined by

(9) vi,j(t+1) ={N(Xbest,j(t)+xi,j(t)2,rand(0,1)≤CR∨j=jrand  |Xbest,j(t)−xi,j(t)|)xi,j(t)otherwise,

where *N* represents a Gaussian distribution with mean (*X*_best,*j*_(*t*) + *x*_*i*,*j*_(*t*))/2 and standard deviation |*X*_best,*j*_(*t*) − *x*_*i*,*j*_(*t*)| and CR is the probability of crossover.

To balance the global search ability and convergence rate, Wang et al. [22] proposed a modified GBDE (called MGBDE). The mutation strategy uses a hybridization of GBDE and DE/best/1 as follows:

(10) vi,j(t+1) ={Xbest,j(t)+F·(xi1,j(t)−xi2,j(t))rand(0,1)≤0.5N(Xbest,j(t)+xi,j(t)2,|Xbest,j(t)−xi,j(t)|)otherwise.

## 4. Proposed Algorithm: BBTLBO

The bare-bones PSO utilizes this information by sampling candidate solutions, normally distributed around the formally derived attractor point. That is, the new position is generated by a Gaussian distribution for sampling the search space based on the *g*_best_ and the *p*_best_ at the current iteration. As a result, the new position will be centered around the weighted average of *p*_best_ and *g*_best_. Generally speaking, at the initial evolutionary stages, the search process focuses on exploration due to the large deviation. With an increasing number of generations, the deviation becomes smaller, and the search process will focus on exploitation. From the search behavior of BBPSO, the Gaussian sampling is a fine tuning procedure which starts during exploration and is continued to exploitation. This can be beneficial for the search of many evolutionary optimization algorithms. Additionally, the bare-bones PSO has no parameters to be tuned.

**Figure 1:**
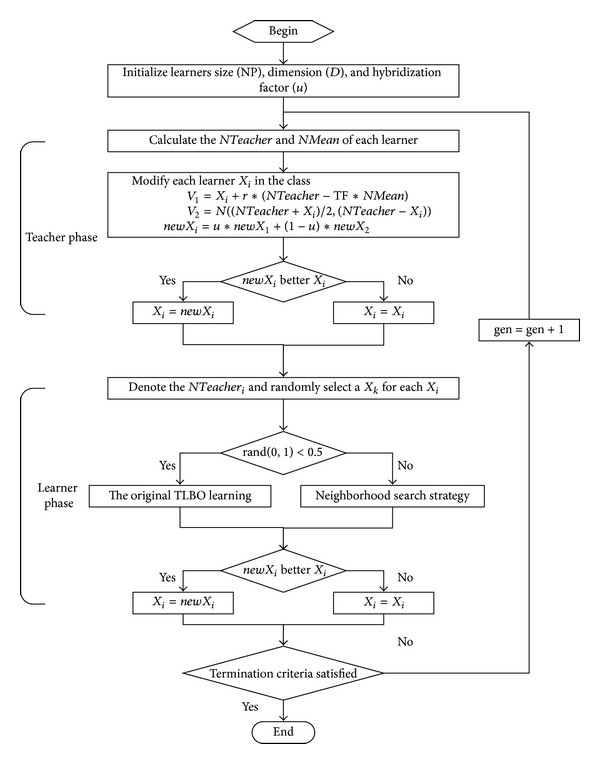
Flow chart showing the working of BBTLBO algorithm.

Based on a previous explanation, a new bare-bones TLBO (BBTLBO) with neighborhood search is proposed in this paper. In fact, for TLBO, if the new learner has a better function value than that of the old learner, it is replaced with the old one in the memory. Otherwise, the old one is retained in the memory. In other words, a greedy selection mechanism is employed as the selection operation between the old and the candidate one. Hence, the new teacher and the new learner are the global best (*g*_best_) and learner's personal best (*p*_best_) found so far, respectively. The complete flowchart of the BBTLBO algorithm is shown in Figure 1.

### 4.1. Neighborhood Search

It is known that birds of a feather flock together and people of a mind fall into the same group. Just like evolutionary algorithms themselves, the notion of neighborhood is inspired by nature. Neighborhood technique is an efficient method to maintain diversity of the solutions. It plays an important role in evolutionary algorithms and is often introduced by researchers in order to allow maintenance of a population of diverse individuals and improve the exploration capability of population-based heuristic algorithms [23–26]. In fact, learners with similar interests form different learning groups. Because of his or her favor characteristic, the learner maybe learns from the excellent individual in the learning group.

**Figure 2:**
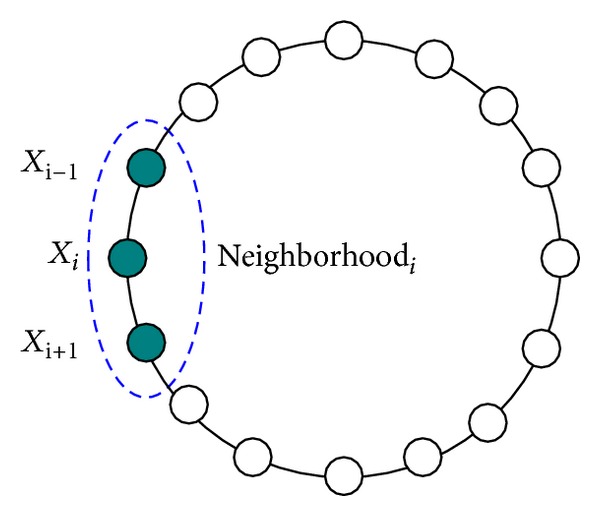
Ring neighborhood topology with three members.

For the implementation of grouping, various types of connected distances may be used. Here we have used a ring topology [27] based on the indexes of learners for the sake of simplicity. In a ring topology, the first individual is the neighbor of the last individual and vice versa. Based on the ring topology, a *k*-neighborhood radius is defined, where *k* is a predefined integer number. For each individual, its *k*-neighborhood radius consists of 2*k* + 1 individuals (including oneself), which are *X*_*i*−*k*_,…, *X*_*i*_,…, *X*_*i*+*k*_. That is, the neighborhood size is 2*k* + 1 for a *k*-neighborhood. For simplicity, *k* is set to 1 (Figure 2) in our algorithm. This means that there are 3 individuals in each learning group. Once groups are constructed, we can utilize them for updating the learners of the corresponding group.

### 4.2. Teacher Phase

To balance the global and local search ability, a modified interactive learning strategy is proposed in teacher phase. In this learning phase, each learner employs an interactive learning strategy (the hybridization of the learning strategy of teacher phase in the standard TLBO and Gaussian sampling learning) based on neighborhood search.

In BBTLBO, the updating formula of the learning for a learner *X*_*i*_ in teacher phase is proposed by the hybridization of the learning strategy of teacher phase and the Gaussian sampling learning as follows:

(11) V1,j(t+1)=Xi,j(t)+rand(0,1)·(NTeacheri,j(t)−TF·NMeani,j(t)),V2,j(t+1)=N(NTeacheri,j(t)+NMeani,j(t)2,|NTeacheri,j(t)−NMeani,j(t)|),newXi,j(t+1)=u·V1,j(t+1)+(1−u)·V2,j(t+1),

where *u* called the hybridization factor is a random number in the range [0, 1] for the *j*th dimension, *NTeacher* and *NMean* are the existing neighborhood best solution and the neighborhood mean solution of each learner, and TF is a teaching factor which can be either 1 or 2 randomly.

In the BBTLBO, there is a (*u*∗100)% chance that the *j*th dimension of the *i*th learner in the population follows the behavior of the learning strategy of teacher phase, while the remaining (100 − *u*∗100)% follow the search behavior of the Gaussian sampling in teacher phase. This will be helpful to balance the advantages of fast convergence rate (the attraction of the learning strategy of teacher phase) and exploration (the Gaussian sampling) in BBTLBO.

### 4.3. Learner Phase

At the same time, in the learner phase, a learner interacts randomly with other learners for enhancing his or her knowledge in the class. This learning method can be treated as the global search strategy (shown in (3)).

In this paper, we introduce a new learning strategy in which each learner learns from the neighborhood teacher and the other learner selected randomly of his or her corresponding neighborhood in learner phase. This learning method can be treated as the neighborhood search strategy. Let *newX*_*i*_ represent the interactive learning result of the learner *X*_*i*_. This neighborhood search strategy can be expressed as follows:

(12) newXi,j=Xi,j+r1∗(NTeacheri,j−Xi,j)+r2∗(Xi,j−Xk,j),

where *r*_1_ and *r*_2_ are random vectors in which each element is a random number in the range [0,1], *NTeacher* is the teacher of the learner *X*_*i*_'s corresponding neighborhood, and the learner *X*_*k*_ is selected randomly from the learner's corresponding neighborhood.

In BBTLBO, each learner is probabilistically learning by means of the global search strategy or the neighborhood search strategy in learner phase. That is, about 50% of learners in the population execute the learning strategy of learner phase in the standard TLBO (shown in (3)), while the remaining 50% execute neighborhood search strategy (shown in (12)). This will be helpful to balance the global search and local search in learner phase.

Moreover, compared to the original TLBO, BBTLBO only modifies the learning strategies. Therefore, both the original TLBO and BBTLBO have the same time complexity *O* (NP · *D* · Gen_max⁡_), where NP is the number of the population, *D* is the number of dimensions, and Gen_max⁡  _ is the maximum number of generations.

As explained above, the pseudocode for the implementation of BBTLBO is summarized in Algorithm 2.

## 5. Functions Optimization

In this section, to illustrate the effectiveness of the proposed method, 20 benchmark functions are used to test the efficiency of BBTLBO. To compare the search performance of BBTLBO with some other methods, other different algorithms are also simulated in the paper.

### 5.1. Benchmark Functions

The details of 20 benchmark functions are shown in Table 1. Among 20 benchmark functions, *F*_1_ to *F*_9_ are unimodal functions, and *F*_10_ to *F*_20_ are multimodal functions. The searching range and theory optima for all functions are also shown in Table 1.

### 5.2. Parameter Settings

All the experiments are carried out on the same machine with a Celoron 2.26 GHz CPU, 2 GB memory, and Windows XP operating system with Matlab 7.9. For the purpose of reducing statistical errors, each algorithm is independently simulated 50 times. For all algorithms, the population size was set to 20. Population-based stochastic algorithms use the same stopping criterion, that is, reaching a certain number of function evaluations (FEs).

### 5.3. Effect of Variation in Parameter *u*

**Figure 3:**
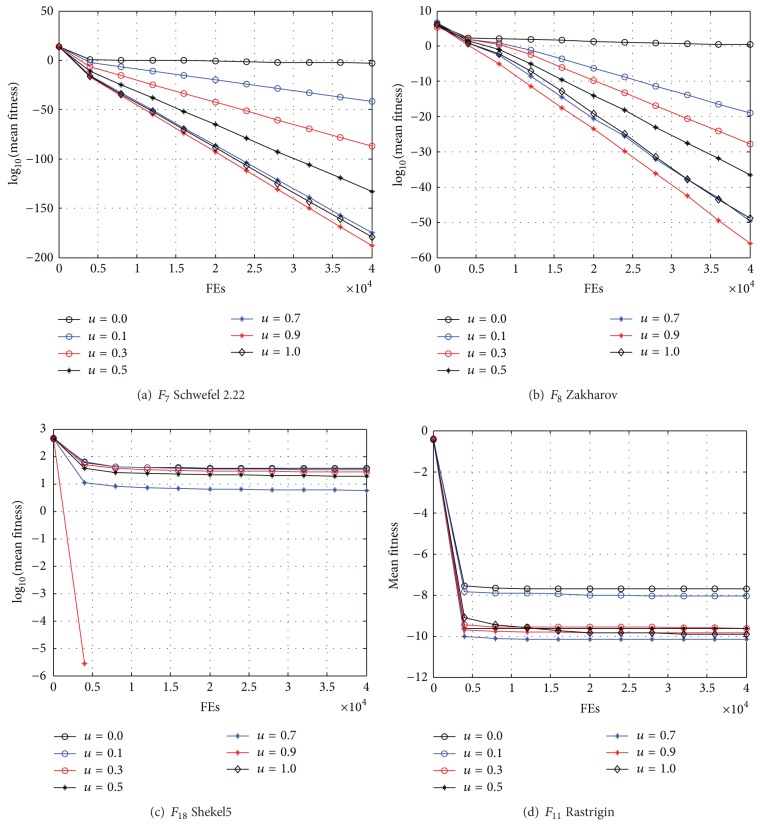
Comparison of the performance curves using different *u*.

The hybridization factor u is set to {0.0,0.1,0.3,0.5,0.7,0.9,1.0}. Comparative tests have been performed using different *u*. In our experiment, the maximal FEs are used as ended condition of algorithm, namely, 40,000 for all test functions.  Table 2 shows the mean optimum solutions and the standard deviation of the solutions obtained using different hybridization factor *u* in the 50 independent runs. The best results among the algorithms are shown in bold.  Figure 3 presents the representative convergence graphs of different benchmark functions in terms of the mean fitness values achieved by using different hybridization factor *u* on all test functions. Due to the tight space limitation, some sample graphs are illustrated.

The comparisons in Table 2 and Figure 3 show that when the hybridization factor *u* is set to 0.9, BBTLBO offers the best performance on 20 test functions. Hence, the hybridization factor *u* is set to 0.9 in the following experiments.

### 5.4. Comparison of BBTLBO with Some Similar Bare-Bones Algorithms

In this section, we compare BBTLBO with five other recently proposed three bare-bones DE variants and two bare-bones PSO algorithms. Our experiment includes two series of comparisons in terms of the solution accuracy and the solution convergence (convergence speed and success rate). We compared the performance of BBTLBO with other similar bare-bones algorithms, including BBPSO [20], BBExp [20], BBDE [21], GBDE [22], and MGBDE [22].

#### 5.4.1. Comparisons on the Solution Accuracy

**Figure 4:**
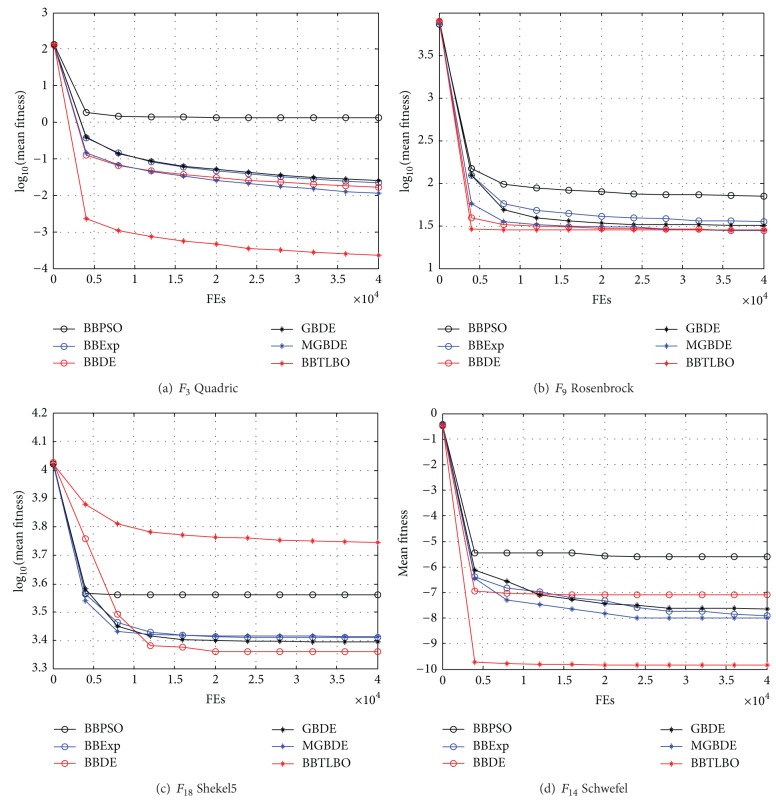
Comparison of the performance curves using different algorithms.

In our experiment, the maximal FEs are used as ended condition of algorithm, namely, 40,000 for all test functions. The results are shown in Table 3 in terms of the mean optimum solution and the standard deviation of the solutions obtained in the 50 independent runs by each algorithm on 20 test functions. The best results among the algorithms are shown in bold.  Figure 4 presents the convergence graphs of different benchmark functions in terms of the mean fitness values achieved by 7 algorithms for 50 independent runs. Due to the tight space limitation, some sample graphs are illustrated.

From Table 3 it can be observed that the mean optimum solution and the standard deviation of all algorithms perform well for the functions *F*_15_ and *F*_17_. Although BBExp performs better than BBTLBO on function *F*_9_ and MGBDE performs better than BBTLBO on function *F*_20_, our approach BBTLBO achieves better results than other algorithms on the rest of test functions. Table 3 and Figure 4 conclude that the BBTLBO has a good performance of the solution accuracy for test functions in this paper.

#### 5.4.2. Comparison of the Convergence Speed and SR

In order to compare the convergence speed and successful rate (SR) of different algorithms, we select a threshold value of the objective function for each test function. For other functions, the threshold values are listed in Table 4. In our experiment, the stopping criterion is that each algorithm is terminated when the best fitness value so far is below the predefined threshold value (*T* Value) or the number of FEs reaches to the maximal FEs 40,000. The results are shown in Table 4 in terms of the mean number of FEs (MFEs) required to converge to the threshold and successful rate (SR) in the 50 independent runs. “NaN” represents that no runs of the corresponding algorithm converged below the predefined threshold before meeting the maximum number of FEs. The best results among the six algorithms are shown in boldface.

From Table 5 it can be observed that all algorithms hardly converge to the threshold for unimodal functions *F*_3_, *F*_5_, *F*_6_, and *F*_8_ and multimodal functions *F*_11_, *F*_12_, and *F*_14_. BBTLBO converges to the threshold except for functions *F*_3_, *F*_9_, and *F*_14_. From the results of total average FEs, BBTLBO converges faster than other algorithms on all unimodal functions and the majority of multimodal functions except for functions *F*_15_, *F*_16_, *F*_19_, and *F*_20_. The acceleration rates between BBTLBO and other algorithms are mostly 10 for functions *F*_1_, *F*_2_, *F*_4_, *F*_7_, *F*_9_, *F*_10_, and *F*_13_. From the results of total average SR, BBTLBO achieves the highest SR for those test functions of which BBTLBO successfully converges to the threshold value. It can be concluded that the BBTLBO has a good performance of convergence speed and successful rate (SR) of the solutions for test functions in this paper.

### 5.5. Comparison of BBTLBO with DE Variants, PSO Variants, and Some TLBO Variants

In this section, we compared the performance of BBTLBO with other optimization algorithms, including jDE [28], SaDE [29], PSOcfLocal [27], PSOwFIPS [30], and TLBO [8, 9]. In our experiment, the maximal FEs are used as the stopping criterion of all algorithms, namely, 40,000 for all test functions. The results are shown in Table 5 in terms of the mean optimum solution and the standard deviation of the solutions obtained in the 50 independent runs by each algorithm on 20 test functions, where “*w*/*t*/*l*” summarizes the competition results among BBTLBO and other algorithms. The best results among the algorithms are shown in boldface.

The comparisons in Table 5 show that that all algorithms perform well for *F*_15_, *F*_16_, and *F*_17_. Although SaDE outperforms BBTLBO on *F*_14_, PSOcfLocal outperforms BBTLBO on *F*_9_ and PSOwFIPS outperforms BBTLBO on *F*_19_ and *F*_20_, and BBTLBO offers the highest accuracy on functions *F*_3_, *F*_4_, *F*_5_, *F*_7_, *F*_8_, *F*_10_, *F*_11_, and *F*_18_. “*w*/*t*/*l*” shows that BBTLBO offers well accuracy for the majority of test functions in this paper. Table 5 concludes that BBTLBO has a good performance of the solution accuracy for all unimodal optimization problems and most complex multimodal optimization problems.

## 6. Two Real-World Optimization Problems

In this section, to show the effectiveness of the proposed method, the proposed BBTLBO algorithm is applied to estimate parameters of two real-world problems.

### 6.1. Nonlinear Function Approximation

**Figure 5:**
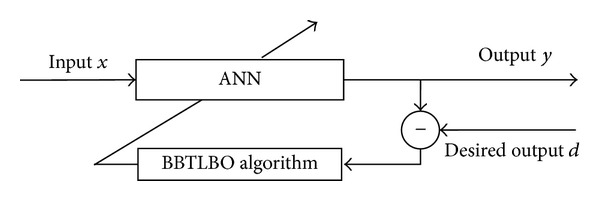
BBTLBO-based ANN.

The artificial neural network trained by our BBTLBO algorithm is a three-layer feed-forward network and the basic structure of the proposed scheme is depicted in Figure 5. The inputs are connected to all the hidden units, which in turn all connected to all the outputs. The variables consist of neural network weights and biases. Suppose a three-layer forward neural network architecture with *M* input units, *N* hidden units, and *K* output units, and the number of the variables is shown as follows:

(13) $$\begin{array}{c} L=\left(M+1\right)*N+\left(N+1\right)*K. \end{array}$$

For neural network training, the aim is to find a set of weights with the smallest error measure. Here the objective function is the mean sum of squared errors (MSE) over all training patterns which is shown as follows:

(14) $$\begin{array}{c} \text{MSE}=\frac{1}{Q*K}\overset{Q}{\underset{i=1}{\sum }}\overset{K}{\underset{j}{\sum }}\frac{1}{2}{\left(d_{ij}-y_{ij}\right)}^{2}, \end{array}$$

where *Q* is the number of training data set, *K* is the number of output units, *d*_*ij*_ is desired output, and *y*_*ij*_ is output inferred from neural network.

In this example, a three-layer feed-forward ANN with one input unit, five hidden units, and one output unit is constructed to model the curve of a nonlinear function which is described by the following equation [31]:

(15) $$\begin{array}{c} y=\sin\left(2x\right)\exp\left(-2x\right). \end{array}$$

**Figure 6:**
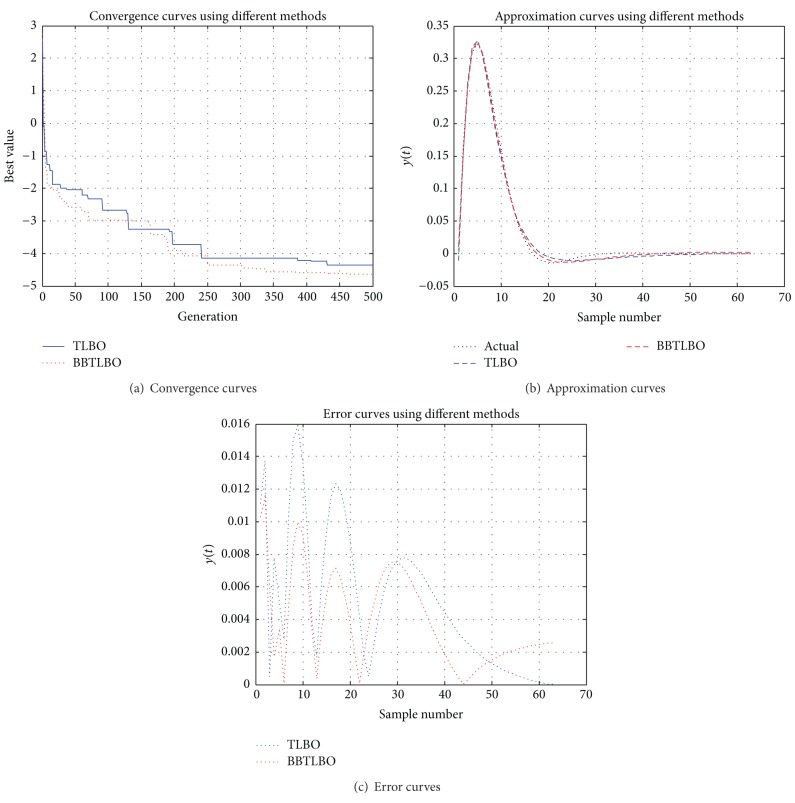
Comparison of the performance curves using different algorithms.

In this case, activation function used in the output layer is the sigma function and activation function used in the output layer is linear. The number (dimension) of the variables is 16 for BBTLBO-based ANN. In order to train the ANN, 200 pairs of data are chosen from the real model. For each algorithm, 50 runs are performed. The other parameters are the same as those of the previous investigations. The results are shown in Table 6 in terms of the mean MSE and the standard deviation obtained in the 50 independent runs for three methods.  Figure 6 shows the predicted time series for training and test using different algorithms. It can conclude that the approximation achieved by BBTLBO has good performance.

### 6.2. Tuning of PID Controller

The continuous form of a discrete-type PID controller with a small sampling period Δ*t* is described as follows [32]:

(16) $$\begin{array}{c} u\left[k\right]=K_{P}\cdot e\left(k\right)+K_{I}\cdot \overset{k}{\underset{i=1}{\sum }}e\left[i\right]\cdot \Delta t+K_{D}\cdot \frac{e\left[k\right]-e\left[k-1\right]}{\Delta t}, \end{array}$$

where *u*[*k*] is the controlled output, respectively. *e*[*k*] = *r*[*k*] − *y*[*k*] is the error signal, *r*[*k*] and *y*[*k*] are the reference signal and the system output, and *K*_*P*_, *K*_*I*_, and *K*_*D*_ represent the proportional, integral and derivate gains, respectively.

For an unknown plant, the goal of this problem is to minimize the integral absolute error (IAE), which is given as follow [32, 33]:

(17) $$\begin{array}{c} f\left(t\right)=\overset{\infty }{\underset{0}{\int }}\left(\omega_{1}\left|e\left(t\right)\right|+\omega_{2}u^{2}\left(t\right)\right)dt+\omega_{3}t_{r}, \end{array}$$

where *e*(*t*) and *u*(*t*) are used to represent the system error and the control output at time *t*, *t*_*r*_ is the rising time, and *ω*_*i*_ (*i* = 1, 2, 3) are weight coefficients.

To avoid overshooting, a penalty value is adopted in the cost function. That is, once overshooting occurs, the value of overshooting is added to the cost function, and the cost function is given as follows [32, 33]:

(18) if  dy(t)<0f(t)=∫0∞(ω1|e(t)|+ω2u2(t)+ω4|dy(t)|)dt+ω3trelsef(t)=∫0∞(ω1|e(t)|+ω2u2(t))dt+ω3trend,

where *ω*_4_ is a coefficient and *ω*_4_ ≫ *ω*_1_, *dy*(*t*) = *y*(*t*) − *y*(*t* − 1), and *y*(*t*) is the output of the controlled objective.

In our simulation, the formulas for the plant examined are given as follows [34]:

(19) $$\begin{array}{c} G\left(s\right)=\frac{1958}{s^{3}+17.89s^{2}+103.3s+190.8}. \end{array}$$

The system sampling time is Δ*t* = 0.05 second and the control value *u* is limited in the range of [−10,10]. Other relevant system variables are *K*_*P*_ ∈ [0,1], *K*_*I*_ ∈ [0,1], and *K*_*D*_ ∈ [0,1]. The weight coefficients of the cost function are set as *ω*_1_ = 0.999, *ω*_2_ = 0.001  *ω*_3_ = 2, and  *ω* = 100 in this example.

In the simulations, the step response of PID control system tuned by the proposed BBTLBO is compared with that tuned by the standard genetic algorithm (GA) and the standard PSO (PSO). The population sizes of GA, PSO, and BBTLBO are 50, and the corresponding maximum numbers of iterations are 50, 50, and 50, respectively. In addition, the crossover rate is set as 0.90 and the mutation rate is 0.10 for GA.

**Figure 7:**
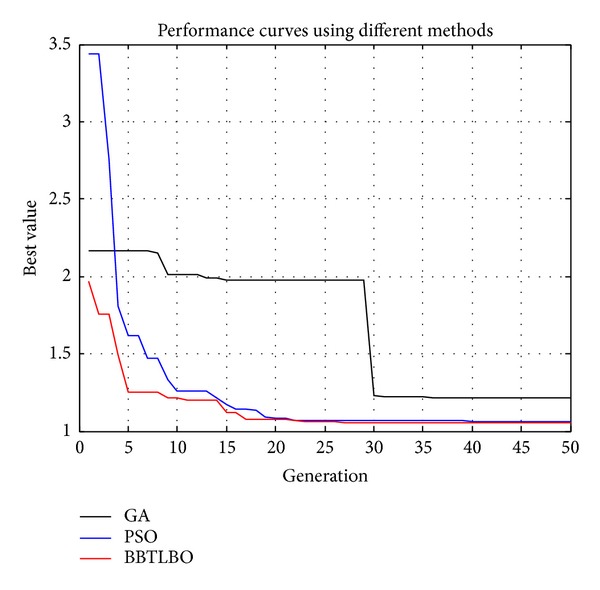
Performance curves using different methods.

**Figure 8:**
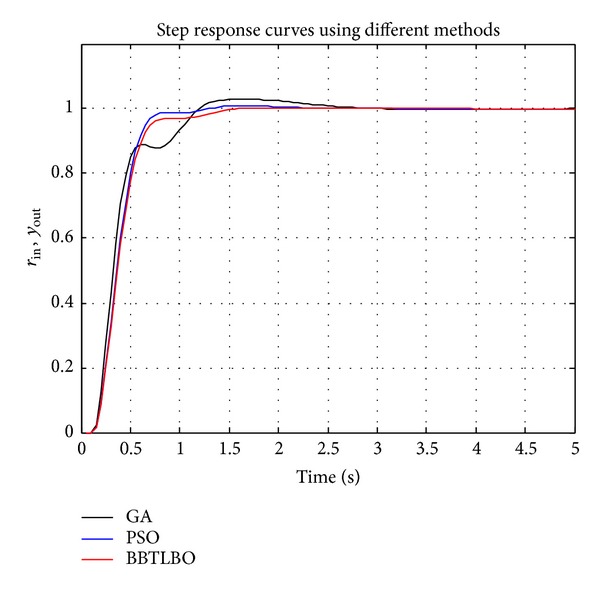
Step response curves using different methods.

The optimal parameters and the corresponding performance values of the PID controllers are listed in Table 7 and the corresponding performance curves and step responses curves are given in Figures 7 and 8. It can be seen from Figure 7 and Table 7 that the PID controller tuned by BBTLBO has the minimum cost function and CPU time. Although PID controllers tuned by PSO have a smaller peak time and rise time, their maximum overshoots are much larger than the overshoot tuned by BBTLBO. It concludes that the PID controller tuned by the BBTLBO could perform the best control performance in the simulations.

## 7. Conclusion

In this paper, TLBO has been extended to BBTLBO which uses the hybridization of the learning strategy in the standard TLBO and Gaussian sampling learning to balance the exploration and the exploitation in teacher phase and uses a modified mutation operation so as to eliminate the duplicate learners in learner phase. The proposed BBTLBO algorithm is utilized to optimize 20 benchmark functions and two real-world optimization problems. From the analysis and experiments, the BBTLBO algorithm significantly improves the performance of the original TLBO, although it needs to spend more CPU time than the standard TLBO algorithm in each generation. From the results compared with other algorithms on the 20 chosen test problems, it can be observed that the BBTLBO algorithm has good performance by using neighborhood search more effectively to generate better quality solutions, although the BBTLBO algorithm does not always have the best performance in all experiments cases of this paper. It can be also observed that the BBTLBO algorithm gives the best performance on two real-world optimization problems compared with other algorithms in the paper.

Further work includes research into neighborhood search based on different topological structures. Moreover, the algorithm may be further applied to constrained, dynamic, and noisy single-objective and multiobjective optimization domain. It is expected that BBTLBO will be used to more real-world optimization problems.

## Figures and Tables

**Algorithm 1:**
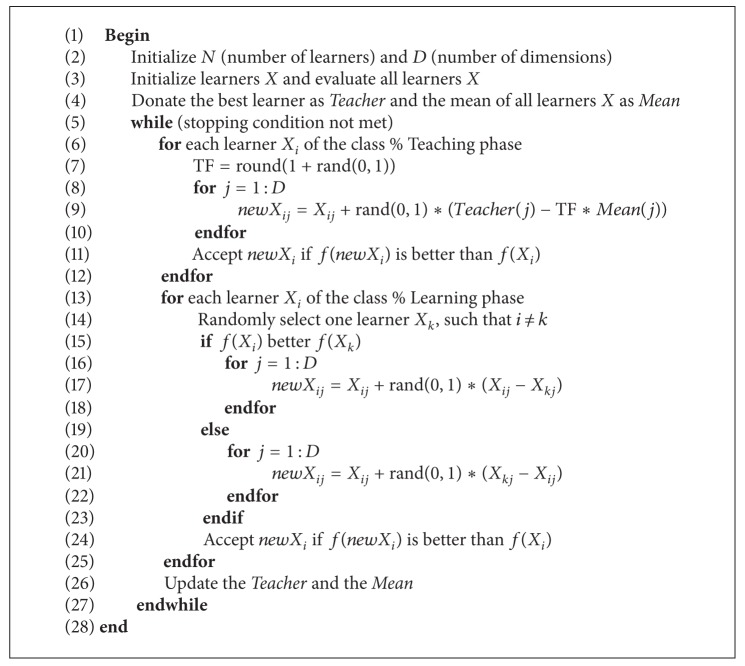
TLBO( ).

**Algorithm 2:**
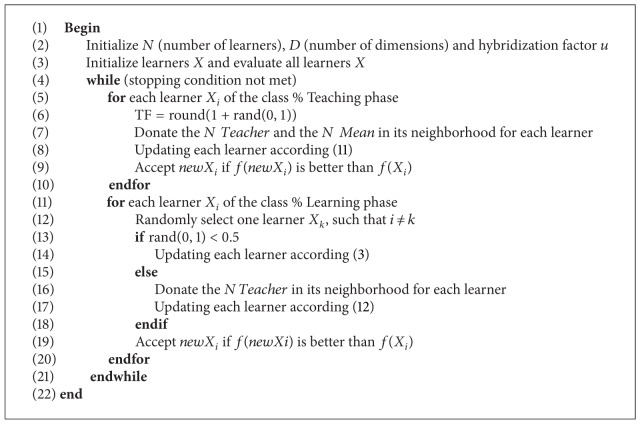
BBTLBO( ).

**Table 1:** Details of numerical benchmarks used.

Function	Formula	*D*	Range	Optima
Sphere	$F_{1}\left(x\right)=\overset{D}{\underset{i=1}{\sum }}x_{i}^{2}$	30	[−100, 100]	0
Sum Square	$F_{2}(x)=\overset{D}{\underset{i=1}{\sum }}ix_{i}^{2}$	30	[−100, 100]	0
Quadric	$F_{3}(x)=\overset{D}{\underset{i=1}{\sum }}ix_{i}^{4}+\text{random}(0,1)$	30	[−1.28, 1.28]	0
Step	$F_{4}(x)=\overset{D}{\underset{i=1}{\sum }}{\left(\left\lfloor x_{i}+0.5\right\rfloor \right)}^{2}$	30	[−100, 100]	0
Schwefel 1.2	$F_{5}(x)=\overset{D}{\underset{i=1}{\sum }}{\left(\overset{i}{\underset{j=1}{\sum }}x_{j}\right)}^{2}$	30	[−100, 100]	0
Schwefel 2.21	$F_{6}(x)=\max\left\{\begin{array}{c} \left|x_{i}\right|,1\leq i\leq D \end{array}\right\}$	30	[−100, 100]	0
Schwefel 2.22	$F_{7}(x)=\overset{D}{\underset{i=1}{\sum }}\left|x_{i}\right|+\prod_{i=1}^{D}\left|x_{i}\right|$	30	[−10, 10]	0
Zakharov	$F_{8}(x)=\overset{D}{\underset{i=1}{\sum }}x_{i}^{2}+{\left(\overset{D}{\underset{i=1}{\sum }}0.5ix_{i}\right)}^{2}+{\left(\overset{D}{\underset{i=1}{\sum }}0.5ix_{i}\right)}^{4}$	30	[−100, 100]	0
Rosenbrock	$F_{9}(x)=\overset{D-1}{\underset{i=1}{\sum }}\left\lfloor 100{\left(x_{i}^{2}-x_{i+1}\right)}^{2}+{\left(x_{i}-1\right)}^{2}\right\rfloor$	30	[−2.048, 2.048]	0
Ackley	$F_{10}(x)=20-20\exp\left(\left(-\frac{1}{5}\right)\sqrt{\left(\frac{1}{D}\right)\overset{D}{\underset{i=1}{\sum }}x_{i}^{2}}\right)-\exp\left(\left(\frac{1}{D}\right)\overset{D}{\underset{i=1}{\sum }}\cos\left(2\pi x_{i}\right)\right)+e$	30	[−32, 32]	0
Rastrigin	$F_{11}(x)=\overset{D}{\underset{i=1}{\sum }}(x_{i}^{2}-10\cos\left(2\pi x_{i}\right)+10)$	30	[−5.12, 5.12]	0
Weierstrass	$\begin{array}{c} F_{12}(x)=\overset{D}{\underset{i=1}{\sum }}\left(\overset{kmax}{\underset{k=0}{\sum }}\left[a^{k}cos\left(2\pi b^{k}\left(x_{i}+0.5\right)\right)\right]\right)-D\overset{k\max}{\underset{k=0}{\sum }}\left[a^{k}\cos\left(2\pi b^{k}\times 0.5\right)\right]\\ \begin{array}{ccc} a=0.5 & b=3 & k\max=20 \end{array} \end{array}$	30	[−0.5, 0.5]	0
Griewank	$F_{13}(x)=\overset{D}{\underset{i=1}{\sum }}\left(\frac{x_{i}^{2}}{4000}\right)-\prod_{i=1}^{n}\cos\left(\frac{x_{i}}{\sqrt{i}}\right)+1$	30	[−600, 600]	0
Schwefel	$F_{14}(x)=418.9829D+\overset{D}{\underset{i=1}{\sum }}\left(-x_{i}\sin\sqrt{\text{abs}\left(x_{i}\right)}\right)$	30	[−500, 500]	0
Bohachevsky1	*F*_15_(*x*) = *x*__1__^2^ + 2*x*_2_^2^ − 0.3cos⁡(3π*x*_1_) − 0.4cos⁡(4π*x*_2_) + 0.7	2	[−100, 100]	0
Bohachevsky2	*F*_16_(*x*) = *x*__1__^2^ + 2*x*_2_^2^ − 0.3cos⁡(3π*x*_1_)∗cos⁡(4π*x*_2_) + 0.3	2	[−100, 100]	0
Bohachevsky3	*F*_17_(*x*) = *x*__1__^2^ + 2*x*_2_^2^ − 0.3cos⁡((3π*x*_1_) + (4π*x*_2_) + 0.3	2	[−100, 100]	0
Shekel5	$F_{18}(x)=-{\overset{5}{\underset{i=1}{\sum }}\left[(x-a_{i}){\left(x-a_{i}\right)}^{T}+c_{i}\right]}^{-1}$	4	[0, 10]	−10.1532
Shekel7	$F_{19}(x)=-{\overset{7}{\underset{i=1}{\sum }}\left[(x-a_{i}){\left(x-a_{i}\right)}^{T}+c_{i}\right]}^{-1}$	4	[0, 10]	−10.4029
Shekel10	$F_{20}(x)=-{\overset{10}{\underset{i=1}{\sum }}\left[(x-a_{i}){\left(x-a_{i}\right)}^{T}+c_{i}\right]}^{-1}$	4	[0, 10]	−10.5364

**Table 2:** Comparisons mean ± std of the solutions using different *u*.

Fun	BBTLBO (*u* = 0.0)	BBTLBO (*u* = 0.1)	BBTLBO (*u* = 0.3)	BBTLBO (*u* = 0.5)	BBTLBO (*u* = 0.7)	BBTLBO (*u* = 0.9)	BBTLBO (*u* = 1.0)
*F*_1_	1.75*e* − 001 ± 1.21*e* + 000	6.89*e* − 071 ± 1.01*e* − 070	1.23*e* − 163 ± 00	1.21*e* − 256 ± 00	0.0 ± 0.0	0.0 ± 0.0	0.0 ± 0.0
*F*_2_	8.98*e* − 005 ± 5.73*e* − 004	5.62*e* − 069 ± 2.72*e* − 068	2.20*e* − 161 ± 1.12*e* − 160	2.43*e* − 254 ± 00	0.0 ± 0.0	0.0 ± 0.0	0.0 ± 0.0
*F*_3_	1.20*e* − 001 ± 6.34*e* − 002	5.91*e* − 003 ± 1.44*e* − 003	1.01*e* − 003 ± 3.48*e* − 004	4.35*e* − 004 ± 1.97*e* − 004	2.35*e* − 004 ± 1.30*e* − 004	2.27*e* − 004 ± 1.26*e* − 004	1.99**e** − 004 ± 1.13**e** − 004
*F*_4_	7.65*e* + 002 ± 5.83*e* + 002	4.80*e* − 001 ± 8.86*e* − 001	0.0 ± 0.0	0.0 ± 0.0	0.0 ± 0.0	0.0 ± 0.0	0.0 ± 0.0
*F*_5_	5.58*e* + 002 ± 6.53*e* + 002	1.87*e* − 028 ± 5.73*e* − 028	3.53*e* − 054 ± 1.86*e* − 053	3.69*e* − 073 ± 2.27*e* − 072	9.53*e* − 096 ± 6.74*e* − 095	2.16**e** − 115 ± 1.10**e** − 114	2.56*e* − 100 ± 1.30*e* − 099
*F*_6_	2.51*e* + 001 ± 5.34*e* + 000	6.67*e* − 021 ± 8.81*e* − 021	2.81*e* − 061 ± 6.36*e* − 061	8.22*e* − 100 ± 1.80*e* − 099	8.18*e* − 137 ± 1.41*e* − 136	3.63**e** − 154 ± 1.34**e** − 153	8.86*e* − 147 ± 3.22*e* − 146
*F*_7_	1.37*e* − 003 ± 9.54*e* − 003	8.72*e* − 043 ± 1.52*e* − 042	5.68*e* − 088 ± 8.76*e* − 088	1.01*e* − 133 ± 2.38*e* − 133	2.60*e* − 175 ± 00	1.16**e** − 188 ± 00	8.33*e* − 180 ± 00
*F*_8_	2.41*e* + 000 ± 3.07*e* + 000	1.32*e* − 019 ± 2.98*e* − 019	2.13*e* − 028 ± 7.69*e* − 028	3.44*e* − 037 ± 1.24*e* − 036	2.20*e* − 050 ± 9.12*e* − 050	1.07**e** − 056 ± 4.39**e** − 056	2.03*e* − 049 ± 8.94*e* − 049
*F*_9_	2.66**e** + 001 ± 1.79**e** + 000	2.72*e* + 001 ± 3.17*e* − 001	2.77*e* + 001 ± 3.18*e* − 001	2.83*e* + 001 ± 2.78*e* − 001	2.84*e* + 001 ± 2.67*e* − 001	2.83*e* + 001 ± 3.41*e* − 001	2.80*e* + 001 ± 3.87*e* − 001
*F*_10_	8.30*e* + 000 ± 1.76*e* + 000	1.77*e* − 001 ± 6.10*e* − 001	5.90*e* − 015 ± 1.70*e* − 015	3.55**e** − 015 ± 00	3.55**e** − 015 ± 00	3.55**e** − 015 ± 00	3.55**e** − 015 ± 00
*F*_11_	3.74*e* + 001 ± 9.05*e* + 000	3.33*e* + 001 ± 1.18*e* + 001	2.71*e* + 001 ± 8.00*e* + 000	1.89*e* + 001 ± 1.14*e* + 001	5.73*e* + 000 ± 1.06*e* + 001	0.0 ± 0.0	0.0 ± 0.0
*F*_12_	8.15*e* + 000 ± 1.93*e* + 000	3.38*e* − 001 ± 1.16*e* + 000	0.0 ± 0.0	0.0 ± 0.0	0.0 ± 0.0	0.0 ± 0.0	0.0 ± 0.0
*F*_13_	5.06*e* − 001 ± 8.08*e* − 001	6.52*e* − 003 ± 8.86*e* − 003	1.78*e* − 003 ± 3.68*e* − 003	0.0 ± 0.0	0.0 ± 0.0	0.0 ± 0.0	0.0 ± 0.0
*F*_14_	4.33**e** + 003 ± 6.79**e** + 002	4.67*e* + 003 ± 6.10*e* + 002	5.17*e* + 003 ± 6.68*e* + 002	5.59*e* + 003 ± 6.85*e* + 002	5.53*e* + 003 ± 7.10*e* + 002	5.58*e* + 003 ± 7.80*e* + 002	5.40*e* + 003 ± 6.53*e* + 002
*F*_15_	0.0 ± 0.0	0.0 ± 0.0	0.0 ± 0.0	0.0 ± 0.0	0.0 ± 0.0	0.0 ± 0.0	0.0 ± 0.0
*F*_16_	0.0 ± 0.0	0.0 ± 0.0	0.0 ± 0.0	0.0 ± 0.0	0.0 ± 0.0	0.0 ± 0.0	0.0 ± 0.0
*F*_17_	0.0 ± 0.0	0.0 ± 0.0	0.0 ± 0.0	0.0 ± 0.0	0.0 ± 0.0	0.0 ± 0.0	0.0 ± 0.0
*F*_18_	−7.71*e* + 000 ± 3.47*e* + 000	−8.06*e* + 000 ± 3.39*e* + 000	−9.64*e* + 000 ± 1.81*e* + 000	−9.65*e* + 000 ± 1.76*e* + 000	−1.02**e** + 001 ± 6.77**e** − 003	−9.85*e* + 000 ± 1.22*e* + 000	−9.93*e* + 000 ± 1.12*e* + 000
*F*_19_	−7.69*e* + 000 ± 3.52*e* + 000	−8.13*e* + 000 ± 3.36*e* + 000	−9.87*e* + 000 ± 1.83*e* + 000	−1.03**e** + 001 ± 9.45**e** − 001	−9.76*e* + 000 ± 1.95*e* + 000	−9.82*e* + 000 ± 1.78*e* + 000	−9.61*e* + 000 ± 1.99*e* + 000
*F*_20_	−8.12*e* + 000 ± 3.53*e* + 000	−9.38*e* + 000 ± 2.69*e* + 000	−1.01*e* + 001 ± 1.65*e* + 000	−1.01**e** + 001 ± 1.61**e** + 000	−9.70*e* + 000 ± 2.28*e* + 000	−9.41*e* + 000 ± 2.43*e* + 000	−1.00*e* + 001 ± 1.69*e* + 000

**Table 3:** Comparisons mean ± std of the solutions using different algorithms.

Fun	BBPSO	BBExp	BBDE	GBDE	MGBDE	BBTLBO
*F*_1_	5.44*e* − 027 ± 1.87*e* − 026	2.62*e* − 024 ± 5.00*e* − 024	3.90*e* − 035 ± 2.00*e* − 034	4.35*e* − 022 ± 1.13*e* − 021	3.35*e* − 035 ± 2.11*e* − 034	0.0 ± 0.0
*F*_2_	13800 ± 2.11*e* + 004	1000 ± 4.63*e* + 003	6.20*e* − 021 ± 4.38*e* − 020	1400 ± 4.52*e* + 003	1.28*e* − 032 ± 8.37*e* − 032	0.0 ± 0.0
*F*_3_	1.32*e* + 000 ± 3.18*e* + 000	2.22*e* − 002 ± 7.55*e* − 003	1.64*e* − 002 ± 9.57*e* − 003	2.49*e* − 002 ± 9.88*e* − 003	1.16*e* − 002 ± 5.26*e* − 003	2.27**e** − 004 ± 1.26**e** − 004
*F*_4_	5.60*e* + 000 ± 9.28*e* + 000	9.60*e* − 001 ± 4.27*e* + 000	7.89*e* + 001 ± 3.05*e* + 002	8.40*e* − 001 ± 9.12*e* − 001	1.08*e* + 000 ± 1.28*e* + 000	0.0 ± 0.0
*F*_5_	1.24*e* + 004 ± 6.66*e* + 003	4.41*e* + 003 ± 3.37*e* + 003	2.09*e* + 000 ± 4.00*e* + 000	5.36*e* + 003 ± 3.26*e* + 003	7.57*e* + 002 ± 1.16*e* + 003	2.16**e** − 115 ± 1.10**e** − 114
*F*_6_	1.67*e* + 001 ± 9.19*e* + 000	1.20*e* + 000 ± 5.22*e* − 001	1.39*e* + 001 ± 4.47*e* + 000	3.60*e* − 001 ± 1.95*e* − 001	1.10*e* + 000 ± 2.94*e* + 000	3.63**e** − 154 ± 1.34**e** − 153
*F*_7_	2.34*e* + 001 ± 1.32*e* + 001	1.00*e* + 000 ± 3.03*e* + 000	4.06*e* − 019 ± 2.15*e* − 018	6.00*e* − 001 ± 2.40*e* + 000	2.00*e* − 001 ± 1.41*e* + 000	1.16**e** − 188 ± 00
*F*_8_	1.87*e* + 002 ± 1.34*e* + 002	1.58*e* + 002 ± 7.00*e* + 001	1.16*e* − 001 ± 2.35*e* − 001	1.72*e* + 002 ± 6.67*e* + 001	2.49*e* + 001 ± 1.99*e* + 001	1.07**e** − 056 ± 4.39**e** − 056
*F*_9_	7.07*e* + 001 ± 1.48*e* + 002	3.57**e** + 001 ± 2.50**e** + 001	2.76*e* + 001 ± 1.06*e* + 001	3.17*e* + 001 ± 2.07*e* + 001	2.76*e* + 001 ± 1.46*e* + 001	2.83*e* + 001 ± 3.41*e* − 001
*F*_10_	1.06*e* + 001 ± 9.29*e* + 000	1.52*e* + 000 ± 5.11*e* + 000	1.34*e* + 000 ± 1.15*e* + 000	2.59*e* + 000 ± 6.45*e* + 000	5.54*e* − 001 ± 2.79*e* + 000	3.55**e** − 015 ± 00
*F*_11_	1.16*e* + 002 ± 3.53*e* + 001	1.81*e* + 001 ± 7.28*e* + 000	6.76*e* + 001 ± 3.89*e* + 001	1.55*e* + 001 ± 5.96*e* + 000	2.03*e* + 001 ± 9.23*e* + 000	0.0 ± 0.0
*F*_12_	2.73*e* + 000 ± 2.11*e* + 000	1.20*e* − 001 ± 4.42*e* − 001	1.73*e* + 000 ± 1.32*e* + 000	1.21*e* − 001 ± 3.37*e* − 001	5.17*e* − 001 ± 8.67*e* − 001	0.0 ± 0.0
*F*_13_	2.14*e* − 002 ± 4.11*e* − 002	2.30*e* − 003 ± 4.29*e* − 003	4.07*e* − 002 ± 4.89*e* − 002	3.08*e* − 003 ± 7.42*e* − 003	4.63*e* − 003 ± 7.16*e* − 003	0.0 ± 0.0
*F*_14_	3.64*e* + 003 ± 6.28*e* + 002	2.58*e* + 003 ± 5.51*e* + 002	2.30*e* + 003 ± 4.09*e* + 002	2.49*e* + 003 ± 5.41*e* + 002	2.60*e* + 003 ± 5.05*e* + 002	5.58**e** + 003 ± 7.80**e** + 002
*F*_15_	0.0 ± 0.0	0.0 ± 0.0	0.0 ± 0.0	0.0 ± 0.0	0.0 ± 0.0	0.0 ± 0.0
*F*_16_	4.37*e* − 003 ± 3.09*e* − 002	0.0 ± 0.0	0.0 ± 0.0	0.0 ± 0.0	0.0 ± 0.0	0.0 ± 0.0
*F*_17_	0.0 ± 0.0	0.0 ± 0.0	0.0 ± 0.0	0.0 ± 0.0	0.0 ± 0.0	0.0 ± 0.0
*F*_18_	−5.60*e* + 000 ± 3.41*e* + 000	−7.90*e* + 000 ± 2.74*e* + 000	−7.09*e* + 000 ± 3.33*e* + 000	−7.63*e* + 000 ± 2.86*e* + 000	−8.01*e* + 000 ± 3.00*e* + 000	−9.85**e** + 000 ± 1.22**e** + 000
*F*_19_	−5.97*e* + 000 ± 3.31*e* + 000	−7.87*e* + 000 ± 3.03*e* + 000	−6.21*e* + 000 ± 3.66*e* + 000	−8.60*e* + 000 ± 2.68*e* + 000	−8.37*e* + 000 ± 2.90*e* + 000	−9.82**e** + 000 ± 1.78**e** + 000
*F*_20_	−5.81*e* + 000 ± 3.65*e* + 000	−9.40*e* + 000 ± 2.42*e* + 000	−6.02*e* + 000 ± 3.77*e* + 000	−9.46**e** + 000 ± 2.24**e** + 000	−9.38*e* + 000 ± 2.51*e* + 000	−9.41*e* + 000 ± 2.43*e* + 000

**Table 4:** The mean number of FEs and SR with acceptable solutions using different algorithms.

Fun	*t* value	BBPSO		BBExp		BBDE		GBDE		MGBDE		BBTLBO	
		MFEs	SR	MFEs	SR	MFEs	SR	MFEs	SR	MFEs	SR	MFEs	SR
*F*_1_	1*E* − 8	15922	**100**	17727	**100**	11042	**100**	19214	**100**	11440	**100**	**1390**	**100**
*F*_2_	1*E* − 8	17515	54	19179	94	12243	**100**	20592	90	12634	**100**	**1500**	**100**
*F*_3_	1*E* − 8	NaN	0	NaN	0	NaN	0	NaN	0	NaN	0	NaN	**0**
*F*_4_	1*E* − 8	11710	24	8120	84	3634	6	7343	40	4704	34	**525**	**100**
*F*_5_	1*E* − 8	NaN	0	NaN	0	NaN	0	NaN	0	NaN	0	**4100**	**100**
*F*_6_	1*E* − 8	NaN	0	NaN	0	NaN	0	NaN	0	NaN	0	**2603**	**100**
*F*_7_	1*E* − 8	17540	6	21191	90	17314	**100**	22684	94	15322	98	**2144**	**100**
*F*_8_	1*E* − 8	NaN	0	NaN	0	NaN	0	NaN	0	NaN	0	**9286**	**100**
*F*_9_	1*E* − 2	17073	62	18404	42	14029	24	18182	52	17200	80	NaN	**0**
*F*_10_	1*E* − 8	24647	26	27598	90	18273	26	29172	82	18320	84	**2110**	**100**
*F*_11_	1*E* − 8	NaN	0	NaN	0	NaN	0	NaN	0	NaN	0	**2073**	**100**
*F*_12_	1*E* − 8	NaN	0	25465	50	NaN	0	27317	64	19704	24	**2471**	**100**
*F*_13_	1*E* − 8	16318	32	21523	58	11048	16	22951	64	14786	58	**1470**	**100**
*F*_14_	1*E* − 8	NaN	0	NaN	0	NaN	0	NaN	0	NaN	0	NaN	**0**
*F*_15_	1*E* − 8	**658**	**100**	1176	100	1274	**100**	1251	**100**	1206	**100**	799	**100**
*F*_16_	1*E* − 8	**657**	98	1251	**100**	1294	**100**	1343	**100**	1308	**100**	813	**100**
*F*_17_	1*E* − 8	995	**100**	2626	**100**	1487	**100**	2759	**100**	1921	**100**	**973**	**100**
*F*_18_	−10.15	1752	34	6720	44	2007	52	4377	32	8113	64	**1684**	**94**
*F*_19_	−10.40	2839	34	8585	48	**1333**	42	6724	50	3056	66	2215	**90**
*F*_20_	−10.53	1190	36	8928	74	**1115**	40	6548	76	5441	80	2822	**82**

**Table 5:** Comparisons mean ± std of the solutions using different algorithms.

Fun	jDE	SaDE	PSOcfLocal	PSOwFIPS	TLBO	BBTLBO
*F*_1_	3.63*e* − 025 ± 1.85*e* − 024	7.65*e* − 025 ± 3.34*e* − 024	9.23*e* − 018 ± 3.03*e* − 017	1.01*e* − 002 ± 5.48*e* − 003	3.05*e* − 189 ± 00	0.0 ± 0.0
*F*_2_	1.49*e* − 023 ± 6.69*e* − 023	2.75*e* − 025 ± 1.08*e* − 024	3.68*e* − 017 ± 5.37*e* − 017	1.08*e* − 001 ± 5.05*e* − 002	1.29*e* − 185 ± 00	0.0 ± 0.0
*F*_3_	3.22*e* − 002 ± 2.83*e* − 002	2.08*e* − 002 ± 1.18*e* − 002	1.28*e* − 002 ± 5.50*e* − 003	1.86*e* − 002 ± 4.39*e* − 003	5.70*e* − 004 ± 2.37*e* − 004	2.27**e** − 004 ± 1.26**e** − 004
*F*_4_	2.11*e* + 001 ± 6.74*e* + 001	0.0 ± 0.0	0.0 ± 0.0	0.0 ± 0.0	0.0 ± 0.0	0.0 ± 0.0
*F*_5_	1.22*e* + 002 ± 1.37*e* + 002	4.28*e* + 001 ± 2.59*e* + 001	1.17*e* + 001 ± 9.30*e* + 000	2.60*e* + 003 ± 6.79*e* + 002	9.45*e* − 043 ± 6.47*e* − 042	2.16**e** − 115 ± 1.10**e** − 114
*F*_6_	3.06*e* + 001 ± 8.50*e* + 000	2.45*e* + 000 ± 2.60*e* + 000	4.67*e* − 001 ± 2.82*e* − 001	2.66*e* + 000 ± 5.58*e* − 001	2.08*e* − 078 ± 4.30*e* − 078	3.63**e** − 154 ± 1.34**e** − 153
*F*_7_	8.28*e* − 019 ± 3.49*e* − 018	5.40*e* − 016 ± 3.81*e* − 015	1.34*e* − 011 ± 1.27*e* − 011	1.70*e* − 002 ± 2.85*e* − 003	3.84*e* − 096 ± 5.53*e* − 096	1.16**e** − 188 ± 00
*F*_8_	2.16*e* + 000 ± 4.16*e* + 000	4.88*e* − 001 ± 5.82*e* − 001	9.60*e* − 002 ± 6.99*e* − 002	5.86*e* + 001 ± 1.70*e* + 001	7.09*e* − 022 ± 4.99*e* − 021	1.07**e** − 056 ± 4.39**e** − 056
*F*_9_	2.49*e* + 001 ± 1.05*e* + 001	2.61*e* + 001 ± 1.07*e* + 000	2.40**e** + 001 ± 1.52**e** + 000	2.65*e* + 001 ± 3.54*e* − 001	2.55*e* + 001 ± 5.01*e* − 001	2.83*e* + 001 ± 3.41*e* − 001
*F*_10_	5.05*e* − 001 ± 7.06*e* − 001	2.07*e* − 001 ± 4.58*e* − 001	1.94*e* − 001 ± 4.56*e* − 001	2.16*e* − 002 ± 4.37*e* − 003	3.62*e* − 015 ± 5.02*e* − 016	3.55**e** − 015 ± 00
*F*_11_	2.03*e* + 000 ± 1.94*e* + 000	3.86*e* + 000 ± 1.97*e* + 000	4.26*e* + 001 ± 1.06*e* + 001	1.15*e* + 002 ± 1.54*e* + 001	1.55*e* + 001 ± 8.09*e* + 000	0.0 ± 0.0
*F*_12_	2.88*e* − 002 ± 1.45*e* − 001	6.50*e* − 002 ± 1.87*e* − 001	7.89*e* − 001 ± 1.03*e* + 000	1.36*e* + 000 ± 7.41*e* − 001	0.0 ± 0.0	0.0 ± 0.0
*F*_13_	1.87*e* − 002 ± 3.58*e* − 002	1.18*e* − 002 ± 1.75*e* − 002	1.16*e* − 002 ± 1.58*e* − 002	1.06*e* − 001 ± 9.93*e* − 002	0.0 ± 0.0	0.0 ± 0.0
*F*_14_	1.93*e* + 002 ± 1.42*e* + 002	1.35**e** + 002 ± 1.26**e** + 002	4.49*e* + 003 ± 8.25*e* + 002	3.96*e* + 003 ± 8.40*e* + 002	4.82*e* + 003 ± 6.86*e* + 002	5.58*e* + 003 ± 7.80*e* + 002
*F*_15_	0.0 ± 0.0	0.0 ± 0.0	0.0 ± 0.0	0.0 ± 0.0	0.0 ± 0.0	0.0 ± 0.0
*F*_16_	0.0 ± 0.0	0.0 ± 0.0	0.0 ± 0.0	0.0 ± 0.0	0.0 ± 0.0	0.0 ± 0.0
*F*_17_	0.0 ± 0.0	0.0 ± 0.0	0.0 ± 0.0	0.0 ± 0.0	0.0 ± 0.0	0.0 ± 0.0
*F*_18_	−9.40*e* + 000 ± 2.10*e* + 000	−9.25*e* + 000 ± 2.30*e* + 000	−7.76*e* + 000 ± 3.42*e* + 000	−9.79*e* + 000 ± 1.44*e* + 000	−9.72*e* + 000 ± 1.42*e* + 000	−9.85**e** + 000 ± 1.22**e** + 000
*F*_19_	−9.85*e* + 000 ± 1.90*e* + 000	−9.87*e* + 000 ± 1.83*e* + 000	−9.24*e* + 000 ± 2.70*e* + 000	−1.04**e** + 001 ± 4.23**e** − 009	−9.22*e* + 000 ± 2.41*e* + 000	−9.82*e* + 000 ± 1.78*e* + 000
*F*_20_	−9.65*e* + 000 ± 2.23*e* + 000	−1.01*e* + 001 ± 1.59*e* + 000	−9.63*e* + 000 ± 2.50*e* + 000	−1.05**e** + 001 ± 1.01**e** − 004	−9.65*e* + 000 ± 2.23*e* + 000	−9.41*e* + 000 ± 2.43*e* + 000
*w*/*t*/*l*	13/3/4	12/4/4	13/4/3	12/4/4	11/6/3	**—**

**Table 6:** Comparisons between BBTLBO and other algorithms on MSE.

Algorithm	Training error		Testing error	
	Mean	Std	Mean	Std
TLBO	9.85*e* − 004	9.26*e* − 004	9.43*e* − 004	9.18*e* − 004
BBTLBO	3.45*e* − 004	2.02*e* − 004	2.76*e* − 004	1.82*e* − 004

**Table 7:** Comparisons of parameters of PID controllers using different algorithms.

Algorithm	*K*_*P*_	K_I_	K_D_	Overshoot (%)	Peak time (s)	Rise time (s)	Cost function	CPU time (s)
GA	0.11257	0.02710	0.28792	2.90585	1.65000	1.05000	16.34555	7.05900
PSO	0.11772	0.01756	0.27737	1.04808	1.65000	0.65000	11.60773	6.91000
BBTLBO	0.11605	0.01661	0.25803	0.34261	1.80000	0.70000	11.34300	7.04500
